# The Interplay of SIRT1 and Wnt Signaling in Vascular Calcification

**DOI:** 10.3389/fcvm.2018.00183

**Published:** 2018-12-18

**Authors:** Francesca Bartoli-Leonard, Fiona L. Wilkinson, Alex W. W. Langford-Smith, M. Y. Alexander, Ria Weston

**Affiliations:** Translational Cardiovascular Science, Centre for Bioscience, Manchester Metropolitan University, Manchester, United Kingdom

**Keywords:** SIRT1, vascular calcification, Wnt, β-catenin, diabetes, calcifying vascular cells

## Abstract

Vascular calcification is a major health risk and is highly correlated with atherosclerosis, diabetes, and chronic kidney disease. The development of vascular calcification is an active and complex process linked with a multitude of signaling pathways, which regulate promoters and inhibitors of osteogenesis, the balance of which become deregulated in disease conditions. SIRT1, a protein deacetylase, known to be protective in inhibiting oxidative stress and inflammation within the vessel wall, has been shown as a possible key player in modulating the cell-fate determining canonical Wnt signaling pathways. Suppression of SIRT1 has been reported in patients suffering with cardiovascular pathologies, suggesting that the sustained acetylation of osteogenic factors could contribute to their activation and in turn, lead to the progression of calcification. There is clear evidence of the synergy between β-Catenin and elevated Runx2, and with Wnt signaling being β-Catenin dependent, further understanding is needed as to how these molecular pathways converge and interact, in order to provide novel insight into the mechanism by which smooth muscle cells switch to an osteogenic differentiation programme. Therefore, this review will describe the current concepts of pathological soft tissue mineralization, with a focus on the contribution of SIRT1 as a regulator of Wnt signaling and its targets, discussing SIRT1 as a potential target for manipulation and therapy.

## Introduction

**Figure 1:**
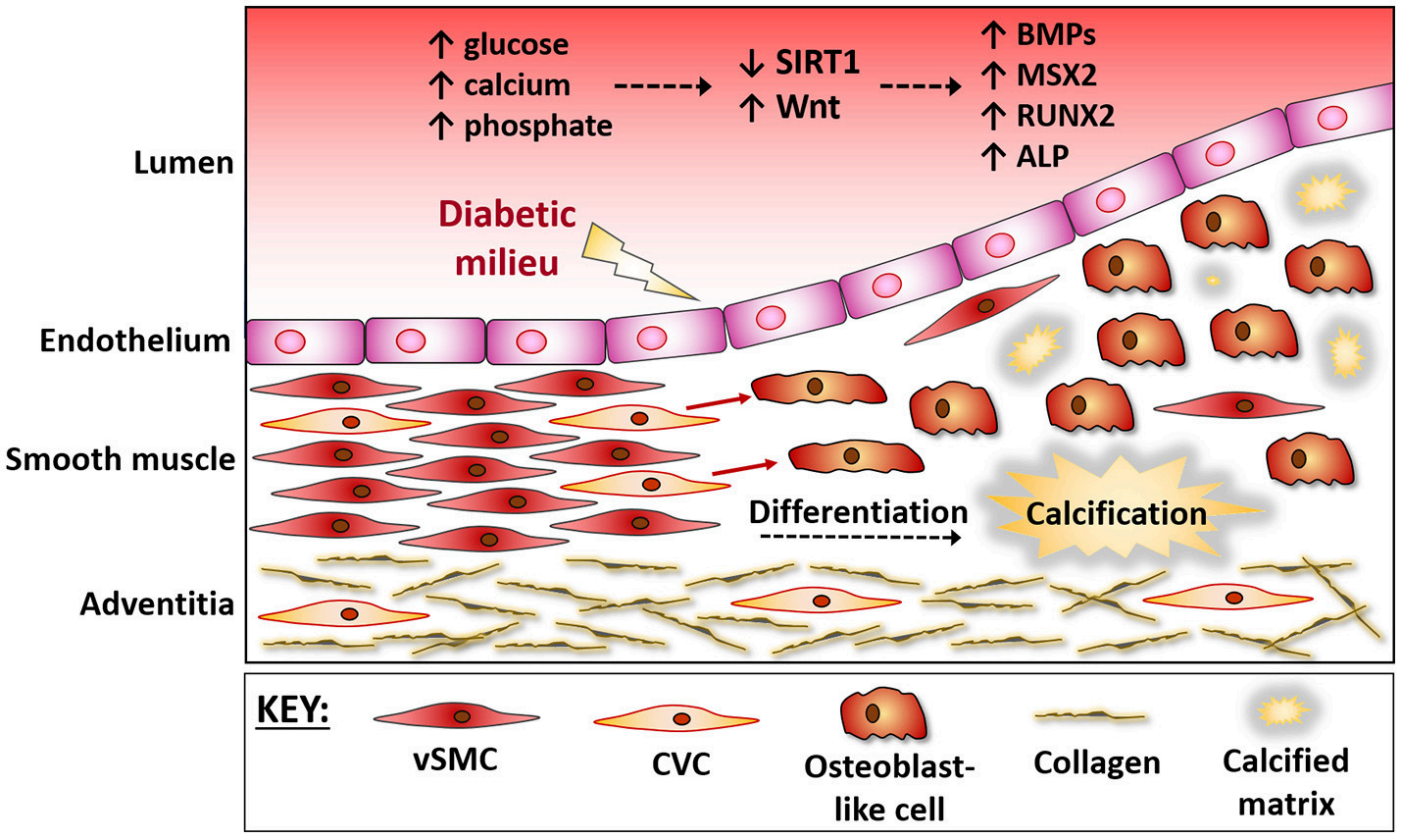
The vessel wall during osteogenic differentiation. The vessel wall responds to the micro-environment within the circulation. During diabetes hyperglycaemia and mineral ion imbalances lead to endothelial damage. The medial layer then responds via triggering a repair response, which often gets masked and further damage ensues. Progenitor cells within the media, often referred to as calcifying vascular cells (CVCs), are believed to up-regulate osteogenic factors and differentiate into bone-forming osteoblasts that contribute to vessel stiffening. These vascular progenitor cells directly sense extracellular signals, including a down regulation of SIRT1 and activation of Wnt signaling, and the protective mechanisms are over-ridden, causing a differentiation of CVCs into bone-forming osteoblasts. BMPs, Bone Morphogenic Proteins; MSX2, msh homeobox 2; RUNX2, Runt related transcription factor 2; OCN, Osteocalcin; CVC, Calcifying Vascular Cells; vSMC, vascular Smooth Muscle Cells.

Vascular calcification is a pathology highly correlated with cardiovascular mortality, and although initially described as Monckeberg's sclerosis (1), with calcium being deposited in the medial layer of arteries, it is now known to be an active process, similar to bone development (2–4). Whilst development of calcification occurs naturally in vessels as they age (5), increased calcification occurs in those with diabetes and chronic kidney disease (CKD), in which constant high plasma glucose and an augmented lipid profile; (comprising of low HDL cholesterol, elevated triglycerides, high LDL cholesterol and high total cholesterol) (1) increases their risk of accelerating calcification development. In healthy tissues, vascular smooth muscle cells (vSMCs) exist within the medial layer of the vessel wall in a quiescent, contractile state, expressing a range of contractile proteins, including smooth muscle α-actin, smooth muscle myosin heavy chain, calponin, and smoothelin. However, in response to these local cues they lose expression of these proteins and gain the capability to transdifferentiate from vSMCs to a more synthetic, osteoblastic phenotype, stiffening, and narrowing the vessel wall (6, 7) (Figure 1). Whilst the synthetic phenotype is thought to possess a protective role, contributing to the deposition of a fibrous cap and thus stabilize an atherosclerotic plaque, the intimal SMCs are believed to be detrimental as they acquire foam cell properties, leading to an inflammatory phenotype (8). Many signaling pathway and transcription factors have been shown to govern the contractile, osteogenic or synthetic features of the vasculature (2, 4, 9–12), and with more understanding of the influence of epigenetics on SMC regulation, their role in the pathogenesis of human vascular disease will only expand (13). *In vitro* models use glucose, calcium and inorganic phosphate as inducers of calcification within vSMCs, with deposition of calcium on the extracellular matrix, and an upregulation of osteogenic markers including alkaline phosphatase (ALP), Runt-relative transcription factor (Runx2), and osteocalcin (14). Calcifying vascular cells (CVCs) are a sub-population of vSMCs susceptible to calcification, which differentiate from stem cell progenitor lineages within the vasculature (15–18). CVCs are characterized as a highly proliferating cell with considerable phenotypic plasticity, where the cells respond to local signals which are activated in disease conditions, including bone morphogenetic proteins (BMPs) and Wnts, and are capable of downregulating contractile proteins and remodeling the extracellular matrix to facilitate migration and differentiation.

Sirtuin 1, (SIRT1) has been identified as a highly conserved nicotinamide adenine dinucleotide-dependent deacetylase, interacting with a range of protein targets involved in Wnt signaling, glucose homeostasis, insulin regulation, and calcium signaling (19), making SIRT1 an attractive candidate for control of calcification. Smooth muscle specific acetylation sites have been identified which allow repression or access to the cellular transcriptional machinery and are regulated via a range of stimuli including transforming growth factor beta (TGF-β), platelet-derived growth factor (PDGF) and oxidized phospholipids, which execute their actions by modulating SMC chromatin structure (20). Wnt signaling and its downstream mediators affect a range of biological processes, first identified in embryonic development (21). The Wnt family is a highly conserved group of 19 genes encoding cysteine-rich-secreted glycoproteins, first identified in *Drosophilia melanogaster* as a mutant wingless gene (22). Subsequent studies demonstrated sequence homology with the *Int-1* gene present in vertebrae and thus the nomenclature *Wnt* was coined in 1991 (23). Being highly conserved and well-studied in eukaryotes, Wnt signaling became recognized as one of the cornerstones for embryonic development, regulating cellular proliferation, polarity, and apoptosis and subsequently becoming suppressed in adults (23). Recent studies have shown a reactivation of Wnt signaling in a variety of cardiovascular pathologies (24, 25), acting as a cell fate determination switch, allowing cellular differentiation to occur, where aberrant Wnt signaling is diverted toward disease progression. This review will discuss the role of SIRT1 in vascular calcification, as well as an overview on Wnt signaling and a summary of potential therapeutic interventions that could modulate osteogenic differentiation, thus linking both SIRT1 and Wnt signaling to vascular calcification.

## Modulation of SIRT1 and Osteogenic Reprogramming

The development of smooth muscle calcification occurs in the presence of hyperphosphatemia, often coupled with hyperglycaemia, in patients with diabetes and CKD. The histone deacetylase SIRT1, known to ameliorate calcification (26), is shown to be decreased in diabetic models (27, 28). The suppression of SIRT1 within blood or tissue allows a build-up of sodium-dependent phosphate co-transporters (29), increasing the concentration of phosphate systemically and within vessels, which is recognized as a key trigger in the development of calcification. Furthermore, diabetic SIRT1 +/– mice exhibited a greater propensity to undergo calcification within the aorta (30). Elevated phosphate within the circulation increases expression of systemic osteogenic and inflammatory factors, activating Wnt signaling and osteogenic transcription factors Msx2 and Runx2 (31). Subsequently, levels of osteocalcin, RANKL, Sclerostin, Osterix, BMPs, and ALP (32, 33) activity are increased. Elevated BMPs form a positive feedback loop, activating the SMAD pathway, sustaining Wnt activation and its downstream targets, Msx2 and Runx2. Runx2 has also been linked to vascular fibrosis, in the absence of overt calcification, highlighting the important role of SIRT1 regulated signals in the multistep processes that control osteogenic programming and calcium deposition (34).

This influx of pro-osteogenic activators coincides with the loss of endogenous contractile vSMC genes such as SM22α and smooth muscle-actin, with a concordant up-regulation of calcification inhibitor proteins including osteopontin in an attempt to counteract the effect. However, under disease conditions the inhibitory effects are masked by the more dominant stimuli and this generally leads to a change in cellular morphology and an increase in extracellular matrix deposition (35). Whilst CVCs have historically been reported to be differentiated vSMCs (36), staining of cells within atherosclerotic plaques have identified both macrophage and stem cell markers, suggesting that CVCs may be sourced from a range of progenitor-like cells within the media or adventitia (18, 37, 38). Recently, SIRT1 has been shown to translocate to the nucleus during neuronal differentiation and repress the Notch3 target Hes1 (39), suggesting a role for SIRT1 during cellular differentiation. Notch3 and Hes1 have also been associated with osteogenic differentiation of vSMCs *in vitro* (9) and mutations in Notch have been shown to lead to aortic valve calcification (40). Therefore, the loss of SIRT1 in diabetes may allow activation of Notch3 and Hes1, leading to osteogenic differentiation of vSMCs and subsequent deposition of a calcified matrix. Whether Wnt, SIRT1, and Notch signaling pathways directly interact, in terms of vSMCs osteogenic differentiation merits further study.

## SIRT1 Suppression and Activation of Canonical Wnt SIGNALING: Driving Factors for Vascular Calcification

**Figure 2:**
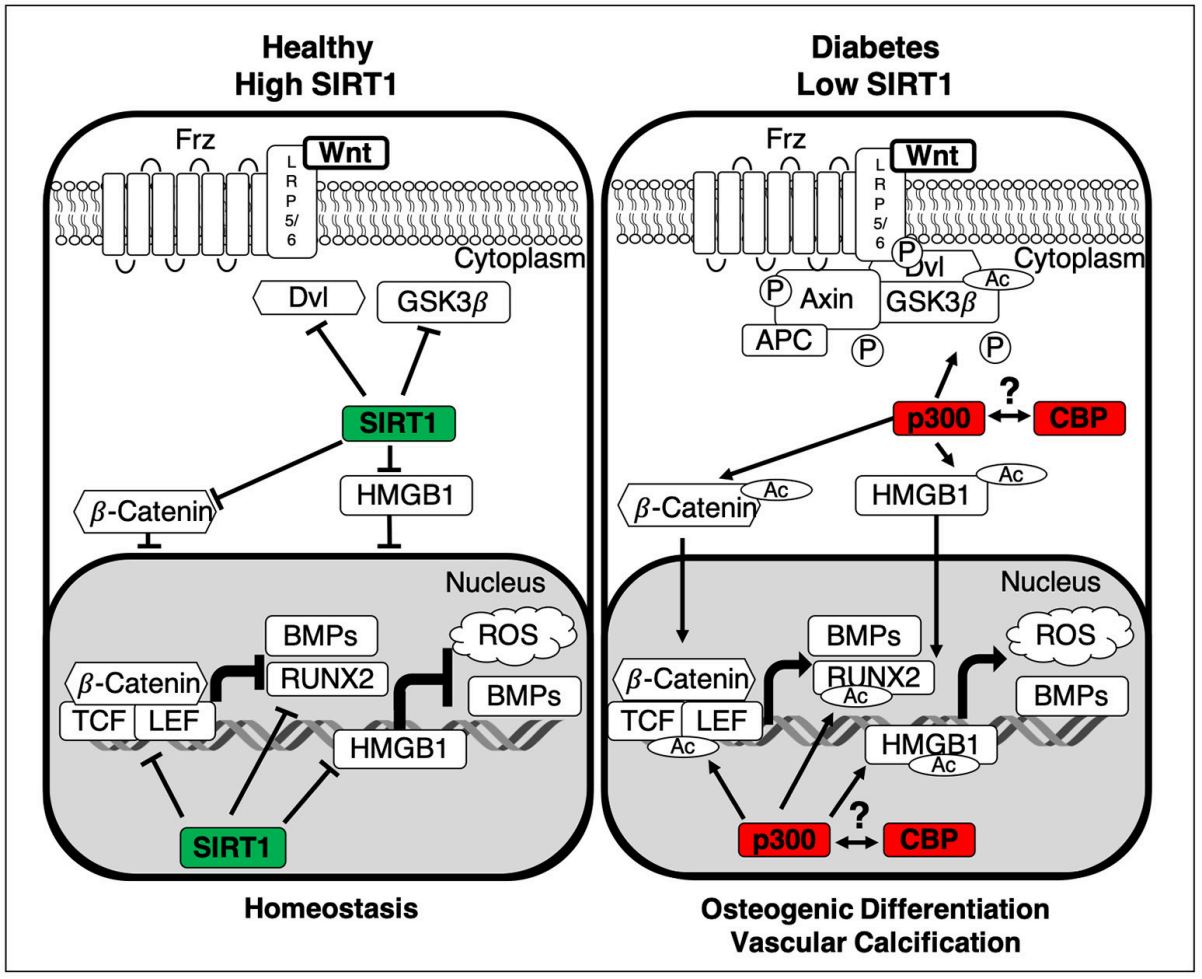
Summary of the interactions between SIRT1 and the canonical Wnt pathway. In healthy homeostatic conditions SIRT1 binds to p300, diminishing its ability to acetylate the β-Catenin complex, comprised of Dvl, GSK3β and β-Catenin. Additionally, SIRT1 deacetylates both β-Catenin and HMGB1 thus inhibiting their translocation to the nucleus, inactivating Wnt signaling and stopping the development of vascular calcification. In the absence of SIRT1 the β-Catenin complex is activated by p300-mediated acetylation and GSK3β-catalyzed phosphorylation, inhibiting its ability to degrade β-Catenin. Additionally, HMGB1 and β-Catenin are also acetylated via p300, facilitating their translocation to the nucleus. Subsequently β-Catenin binds cofactors TCF/LEF and following their acetylation transcription of osteogenic factors Runx2 and BMPs is induced. Whilst the epigenetic roles of CBP and p300 are distinct, the role of CBP still remains an area for further investigation. LRP5/6, Low-density lipoprotein Receptor-related Protein 5/6; APC, Adenomatous Polyposis Coli; GSK3β, Glycogen Synthase Kinase-3 beta; Dvl, Dishelved Protein; TCF/LEF, T-Cell Factor/Lymphoid enhancer factor; Runx2, Runt-Related Transcription Factor; BMPs, Bone Morphogenic Proteins; HMGB1, High Mobility Group Box 1; ROS, Reactive Oxygen Species; Ac, Acetylation; P, Phosphorylation.

Wnt signaling is controlled by the Wnt ligand; a 350 residue hydrophobic protein with a post-translational fatty acid O-acylation modification (41), which is essential for secretion and signal propagation. Post translational modification of Wnt occurs in the endoplasmic reticulum (42) before shutting to the golgi and exportation to the extracellular space (43). Secretion of Wnt is tightly regulated via the wntless transmembrane protein, allowing both paracrine and autocrine activation of the Wnt pathways (44). Activation of Wnt triggers one of three different pathways, all dependent on the Wnt protein firstly becoming palmitoylated on conserved serine residues, then glycosylated and binding to a frizzled cell membrane receptor, propagating signaling within the cell via Disheveled (45–47) (Figure 2). The canonical pathway, involving β-Catenin, has the strongest links to cardiovascular disease and vascular dysfunction (48). Classically, Wnts have been characterized into pro and anti-osteogenic factors, where Wnts such as Wnt5a (49) and Wnt3a are osteogenic, whereas Wnt1 is anti-osteogenic, enforcing a contractile phenotype (46). However, recent work has demonstrated a more complex pathway, in which not only does the active Wnt ligand dictate cell fate, but its interaction with the co-factors and cell surface receptors dictate the emerging osteogenic profile.

Activation of the canonical pathway is characterized by the accumulation of β-Catenin in the cytoplasm and subsequent translocation to the nucleus. When inactive, β-Catenin is constantly degraded via the β-Catenin complex, firstly phosphorylated (50, 51) then ubiquitinated and degraded (52). Conversely when Wnt activates the canonical pathway by binding the transmembrane Frz protein complex (53) it disrupts the β-Catenin complex (54) allowing its translocation to the nucleus (53–55). Without ubiquitination β-Catenin becomes acetylated (56), potentially facilitated by the loss of SIRT1, and binds to target gene promotor elements, triggering increased transcription of a range of osteogenic genes (48). Whilst the role of β-Catenin in diabetes is still illusive, there is clear evidence that hyperglycaemia increases nuclear accumulation of β-Catenin, creating an osteogenic environment. Chronic hyperglycaemic conditions are also shown to displace and inactivate SIRT1 from within the nucleus, allowing acetylation of β-Catenin via p300 (57), promoting the glucose-dependent amplification of Wnt-dependent transcription of osteogenic factors. However, the p300 related acetyltransferase CBP is also engaged in SIRT1 regulatory circuits, including the HIF pathway, which is central in bone formation, Wnt signaling, and vascular calcification. It is increasingly clear that the epigenetic roles of p300 and CBP are distinct, and the role of SIRT1 and CBP in vascular Wnt signaling remains to be elucidated (58–60).

Whilst Wnt signaling is a recognized orchestrator of vascular development within embryos, increasing neovascularisation and proliferation (61) it has been shown to be cell type dependent, with SIRT1 demonstrating varied responses under normal and disease conditions (47). SIRT1 knock-out (62) and knock-in mice (63) clearly demonstrate that a reduction of SIRT1 activates Wnt/β-Catenin signaling (64, 65) and loss of SIRT7 enhances osteogenesis of mesenchymal stem cell differentiation via Wnt signaling (66, 67), both of which contribute to osteogenic differentiation. Therefore, it seems reasonable to suggest that Wnt may be involved in cell fate determination of vSMCs, stimulating their switch to an osteoblastic-like phenotype, and increasing calcified matrix deposition.

BMPs belong to the TGF-β superfamily and are responsible for orchestrating much of the tissue architecture throughout the body (68). Whilst BMPs are key regulators of bone ossification (69) the relationship between BMPs, SIRT1, Wnt signaling, and vascular calcification remains unclear regarding which acts as the initial trigger, or whether they act in concert (70, 71). BMPs are secreted throughout the vessel wall, predominately by pericytes and endothelial cells in a paracrine manner (46) and by vSMCs in an autocrine loop (72). Of the twenty BMPs discovered, BMP2 (73), BMP4 (74), and BMP9 (75) are the most widely reported inducers of bone ossification and vascular calcification in both *in-vitro* and *in-vivo* models. The BMP signaling pathway is activated when BMP binds serine/threonine receptor kinases BMPRI and BMPRII, causing them to heterodimerise and phosphorylate one another (76). Once bound, BMPRI/II phosphorylate receptor-regulated SMADs (R-SMADs) (77, 78), which subsequently bind with co-SMAD4, allowing gene specific promotor binding and osteogenic gene expression (79).

Whilst elevated BMP2 independently induces calcification (80), when combined with β-Catenin activation, the effect is synergistic, increasing mineralized matrix deposition (71, 81). BMP2 is upregulated by hyperglycaemia (82), activating the ligand high mobility group box 1 (HMGB1), which translocates to the nucleus, binding to a cAMP response element (CRE) region of the BMP2 promoter, inducing its expression (83). In contrast, deacetylation of HMGB1 via SIRT1, prevents upregulation of BMP2 (84) (Figure 2), reducing induction of inflammatory markers, including TNFα and reactive oxygen species (85), thus limiting an inflammatory environment conducive to calcification progression. Hyperphosphatemia stimulates BMP2 production, which in turn increases Pit-1 co-transporter expression throughout the cell membrane, allowing an influx of phosphate ions (86). Increased phosphate can downregulate SIRT1 production (28), allowing sustained acetylation of downstream proteins and facilitating hyperacetylation of β-Catenin (87) and Runx2 (88) via p300, thus accelerating calcification.

Although activation of BMP in adults is associated with increased cardiovascular risk and the development of calcification, BMPs have been shown to mediate increased vascular development during embryogenesis, via β-Catenin activation (89). Wnt also controls the development and stability of newly formed bones (90), and as only a proportion of vSMCs calcify within the vessel, it may be suggested that this process is in overdrive by the reactivation of the CVCs, causing bone-like development to form along vessel walls. Downstream of BMPs, phosphorylation of Smad proteins dictates the duration and efficacy of osteogenic gene expression (91). Degradation of β-Catenin, coupled with the inhibition of Smad signaling (79), and prevention of the subsequent signaling pathways impedes the progression of osteogenic differentiation of vSMCs. However, activation and binding of BMPs to their cognate receptor, allows Smad phosphorylation and acetylation via p300 (91), inhibiting Smad ubiquitination (79) and thus propagating the activation of downstream osteogenic genes (92). The suppression of SIRT1 in CVD patients, leads to a reduction in competitive inhibition of p300, thereby increasing BMP/SMAD/p300 signaling and subsequent osteogenic gene transcription.

Human T Cell/Lymphoid Enhancing-Factor (TCF/LEF) (93) regulates cell fate markers, including Wnt, by binding β-catenin alongside co-factors within the nucleus at the TCF response element, to govern gene expression (92), thus causing the progression of vascular calcification. Multiple TCF/LEF response binding elements are present throughout the promotor region of the BMP2 gene (94) which, when bound by a β-Catenin/TCF complex, increases BMP2 transcript production. Inhibition of this activation is achieved via endogenous inhibitor Noggin, suggesting that a positive feedback loop exists between the activation and inhibition of β-Catenin and TCF binding onto the BMP2 promotor (72). Modulation of these proteins within individuals may be the missing link to distinguish between patients more susceptible to calcification and those who appear to be protected.

## Wnt and Runx2: Key Regulators of Osteogenic Differentiation

Runx2; a key osteoblastic transcription factor (95), is essential for chondrocyte maturation and osteogenic differentiation. Originally thought to be expressed solely during bone development, it is now known to be activated in both the intimal and medial layers of the vasculature during calcification development. The activation of Runx2 triggers expression of a range of downstream osteogenic effectors, leading to the differentiation of vSMCs from a contractile to an osteogenic phenotype in a diabetic environment. Hyperphosphatemia downregulates the expression of secreted frizzled-related proteins (SFRPs) (96), which act as a decoy for Wnt signaling, inhibiting internalization of phosphate transporters (97), thereby facilitating the constant entry of phosphate into the cells and propagating the development of calcification. SIRT1 has been shown to regulate SFRPs via deacetylation, directly contributing to their aberrant epigenetic silencing within histone 3 and 4 (47).

Sclerostin, a selective inhibitor of Low-density lipoprotein Receptor-related Protein (LRPs), a member of the Frz membrane complex downstream of Wnt, is increased during CVC differentiation (98). Increased activation of Sclerostin decreases Wnt signal propagation via competitive inhibition, temporarily halting calcification until phosphate and calcium build up within the serum, reactivating the Wnt pathway and further calcifying the vessel. β-Catenin nuclear translocation induces Runx2 expression via SMAD activation in vSMCs (99), binds to the proximal region of the SOST promotor, activating osteogenic transcription, similar to that which occurs in bone (100). Runx2 together with Osterix (Osx) may actively limit the expression of Sclerostin, with polymorphic variations of Runx2 transcriptionally regulating Sclerostin expression in a negative feedback loop (101). Additionally, SIRT1 may decrease SOST gene expression in bone via the deacetylation of histone 3 at lys9 within the promotor region, inhibiting the mechanical loading and subsequent transcription (102). With the development of vascular calcification considered the paradox of bone development, the reduction of SIRT1 in the CVD patient may lead to a decrease in SOST, perpetuating Wnt signaling and vascular calcification.

Runx2 expression is controlled by Wnt signaling via the direct binding of TCF/LCF co-transcription factors. Direct β-Catenin binding to TCF/LEF at the Runx2 promotor site is enhanced by p300 acetylation (23), propagating the Runx2 signaling pathway. Additionally, indirect DNA binding of SMADs and TCF via protein-protein interaction enhances Runx2 expression (57) alongside production of downstream osteogenic proteins. β-Catenin binding to TCF1 at TB1 and TBE2 sites within the Runx2 promotor region has been shown to increase endogenous Runx2 expression 10–20-fold, with damage to these sites attenuating selective Runx2 expression (99). It is clear that vascular calcification involves a network of signaling pathways, not restricted to SIRT1, Runx2, and Wnt and that further studies involving analysis of vSMCs harvested from relevant patient groups could provide insight into the profile of vSMCs during early and late osteogenic differentiation, offering stratified treatment plans to combat calcification development.

## SIRT1 Modulators as Future Calcification Therapies

Activation of Sirtuins, in particular SIRT1, has been shown to decrease Wnt signaling and reduce the risk of cardiovascular disease in both animal and human models (103–105), and administration of resveratrol has been shown to reduce arterial calcification, in both non-human primates and in uremic rats (106, 107) suggesting SIRT1 may be a good candidate molecule for sustained control of vascular calcification. SIRT1 regulates metabolic pathways including 5'AMP and canonical Wnt signaling, both of which underpin key biological events, such as proliferation and differentiation. Developmental studies have demonstrated the crucial role of β-Catenin signaling in bone development, with Wnt-floxed mice developing spontaneous fractures and an inability to develop mature osteoblasts (108). Additionally, the fate of resident stem cells appears to be regulated via the upregulation of the SIRT1/Wnt/β-Catenin pathway, in which MSCs undergo an osteogenic differentiation programme (109, 110). Taken together, it can be suggested that CVCs are part of a resident stem cell population not dissimilar to bone, responding to the absence of SIRT1 and the upregulation of Wnt, causing bone to develop within the vasculature. Bone deposition in the vessel wall is thought to occur in conjunction with bone loss, where calcium and mineral is released from bone, and taken up in the vessel wall, inducing aberrant signaling pathways that result in an osteogenic differentiation of progenitor cells residing in the vessel wall. With the close links between SIRT1 and Wnt signaling, and the activation of Wnt signaling in driving an osteogenic differentiation program by vSMCs (99), understanding Wnt signaling-related interactions with SIRT1 will add insight into the pathogenesis of vascular calcification and enable the development of anti-calcification strategies. Thus, in a pathology where vSMCs are thought to undergo an aberrant differentiation programme, further understanding of SIRT1 signaling in this context, could allow the development of SIRT1 modulators for prevention of this debilitating process (111–113).

Sirtuin activators have generally been described for SIRT1, and resveratrol, a natural compound found as a constituent of grapes and red wine (114), is the most commonly used activator of SIRT1. Resveratrol has been shown to cause resistance to oxidative stress and inflammation and is used widely in the diabetic and age-related decline in heart function and neuronal loss (115, 116). However, because of its modest bioavailability, resveratrol has been reformulated with more efficient small molecules being designed commonly known as synthetic sirtuin activating compounds (STACs) (105, 117), (resVida, Longevinex®, SRT501) (106, 107). Furthermore, molecules that are structurally unrelated to resveratrol (SRT1720 and SRT2379) have been also developed with increased potency, although most have not yet reached the clinic (118, 119). SRT2104 was used recently in a small group of diabetic patients (NCT01031108). It was well-tolerated, but had little beneficial effect on a range of measures of cardiovascular health. However, the study was small, not focussed on human vascular calcification and further investigations will be required to confirm any enhanced metabolic effects (120).

## Concluding Remarks

With an increasingly aging population throughout the Western world, vascular calcification has become a major health concern, correlating with cardiovascular disease development and mortality. Although, the molecular mechanism underpinning vascular calcification remains closely linked to bone formation, the association between loss of SIRT1, activation of Wnt signaling and the upregulation of major osteogenic factors add to the growing armament of deranged osteogenic signaling pathways occurring under pathological conditions in the vessel wall. Additionally, SIRT1, a longevity factor and deacetylase, may act at an epigenetic level to control these converging pathways and cardiovascular risk factors. Furthermore, SIRT7 and SIRT1 have been shown to co-ordinately enhance Sp7/Osx activity and support orthotropic bone formation, and thus further studies could add to the understanding of the role of osteotropic signals during mineralization in skeletal and vascular environments. Indeed, it may be, that other members of the sirtuin family, may contribute to cardiovascular Wnt signaling (121) and either osteoporosis or vascular calcification.

Although the hyperglycaemia and hyperphosphatemia present in many CVD patients is known to suppress SIRT1 expression, there is a growing need for a comprehensive single-cell differentiation pathway for vSMC phenotypic switching, to identify the temporal activity of relevant signaling pathways, and the importance of their early inhibition and reactivation at later time-points. Only then, will we be able to extend our current knowledge of osteogenic vSMC differentiation, which potentially could have implications for future research and clinical application in this field.

## Author Contributions

All authors listed have made a substantial, direct and intellectual contribution to the work, and approved it for publication.

### Conflict of Interest Statement

The authors declare that the research was conducted in the absence of any commercial or financial relationships that could be construed as a potential conflict of interest.
